# An Analysis of the #CovidPain Tweet Chat During the First Wave of the COVID-19 Pandemic in 2020

**DOI:** 10.7759/cureus.18871

**Published:** 2021-10-18

**Authors:** Andrea D Furlan, Zoha Deldar, Linor Berezin, Hance Clarke, Billie Jo Bogden, Daniel Z Buchman

**Affiliations:** 1 Department of Musculoskeletal Rehabilitation, University Health Network - Toronto Rehabilitation Institute, Toronto, CAN; 2 Research, Institute for Work & Health, Toronto, CAN; 3 Department of Medicine, University of Toronto, Toronto, CAN; 4 Biomedical Sciences, Université du Québec à Trois-Rivières, Trois-Rivieres, CAN; 5 Department of Anesthesia and Pain Management, Toronto General Hospital, Toronto, CAN; 6 Patient Partner & Advocate living with chronic pain, N/A, Ottawa, CAN; 7 Clinical Public Health Division, University of Toronto – Dalla Lana School of Public Health, Toronto, CAN

**Keywords:** patient partners, covid-19, pain, social media, twitter

## Abstract

Introduction: In March 2020, we organized two tweet chats to discuss the COVID-19 pandemic and its impact on people affected by chronic pain. The objective of this study is to evaluate the #CovidPain tweet chat activities that took place at the early stages of the COVID-19 pandemic.

Methods: We performed a quantitative analysis of the magnitude, range, engagement, and sentiment of each tweet chat. The data was extracted from Twitter and analyzed in Twitter Analytics and Symplur Signals using frequency and distributions. Then, we conducted a qualitative content analysis of the narrative tweets generated in response to the questions posted during the tweet chats.

Results: The two tweet chats attracted 2305 participants, which generated 4351 tweets. The participants were healthcare providers, patient advocates, researchers/academics, and caregivers. COVID-19 had both negative and positive impacts. The negative consequences of COVID-19 included the reduction of physical activity, canceled appointments and treatments, more isolation, deterioration of preexisting mental health problems, and economic consequences. The positive consequences included efficient use of telemedicine, innovative methods for self-management, and at-home interventions.

Conclusion: Twitter and tweet chats are useful in involving a diverse group of stakeholders for taking a deep dive into the topical issues relevant to a community that might be disproportionately affected by a public health crisis.

## Introduction

The COVID-19 pandemic has the potential to affect people with chronic pain for various reasons, including concerns regarding the condition itself, the shutdown of services provided in communities, the disruption of medication supply chains, burdening the healthcare system, the financial impact to society, social isolation, and the possible risk of illness or death. People with chronic pain seek information through social media platforms such as blogs, Twitter, and YouTube or participating in group discussions. Twitter is a popular social media microblogging platform that uses short messages (280 characters) and has around 300 million active users per month, with around 500 million tweets per day [1,2]. A recent systematic review of media communication on antibiotics and antimicrobial resistance found that social media (Twitter or Facebook) was a means of health information sharing in 32% of the studies, second only to print media with 53% of the studies [3].

Tweet chats are real-time discussions among Twitter users where a moderator posts questions during a preestablished duration of time and people respond to tweets using a unique hashtag. They provide an opportunity to quickly access the opinions of large groups of people around the world, an appropriate method of outreach given the social distancing and travel restrictions in place due to COVID-19. Content mining of tweets has previously been shown to aid in addressing challenges in various areas of healthcare and research, such as health promotion [4], breast cancer survivors [5], and buprenorphine initiation in the emergency department [6].

The principal author (AF) had previous experience organizing tweet chats for other topics prior to the COVID-19 pandemic. The idea of a tweet chat around the impact of COVID-19 on patients living with chronic pain was originally raised by AF and discussed with the coauthor who is a person with lived experience in chronic pain (BJB). Then, the date was selected; more people were invited as moderators (ZD), and representatives of the Canadian Pain Society were involved in assisting in the recruitment and advertising. The hashtag was invented by AF, and it was verified that this hashtag was original and had not been used for other purposes.

This study aims to evaluate the #CovidPain tweet chat activities that took place during the early stages of the COVID-19 pandemic in 2020 with the purpose of assessing their reach and impact.

## Materials and methods

Study procedures

On March 18, 2020, the principal investigator (AF) organized a tweet chat to discuss the impact of the COVID-19 pandemic among the pain community. At the request of various participants from the first tweet chat, a second tweet chat was organized on March 25, 2020. The Canadian Pain Society retweeted the invitations to help recruit participants. AF was the main moderator for the tweet chats and invited three other members of the pain community in Canada to moderate the conversation.

This study is a retrospective analysis of the publicly available platform Twitter. This study was approved by the University Health Network Research Ethics Board (CAPCR/UHN REB #: 20-5405). The population included in this study consisted of Twitter users who attended one or both of the tweet chats on March 18 and 25, 2020. The tweet chats were advertised on Twitter for Canadians living with chronic pain, healthcare providers, researchers, and anyone interested in the topic. The language of both tweet chats was English. The questions were agreed upon among the moderators of each tweet chat prior to the event. Each question was released by AF (@adfurlan) at regular intervals (Table 1). Our study included an analysis of the publicly available data on Twitter using the freely available Twitter analytics and Symplur Signals, a database accessible only after paying a fee.

**Table 1:** Questions and timing posted on each tweet chat *T1Q1 is repeated as T2Q3 to assess if participants became aware of a Health Canada Policy that was released on March 19, 2020 (Health Canada). #Distraction question

T: Tweet chat (date)	Q: Questions
Tweet chat 1 (March 18, 2020)	Question 1*: How is COVID-19 affecting prescriptions and refills of pain medications?
	Question 2: How is COVID-19 affecting the mood of people with chronic pain?
	Question 3: How is COVID-19 affecting physical activity among people with chronic pain?
	Question 4: How do viral infection symptoms (fever, muscle aches) affect people with chronic pain?
	Question 5: How does COVID-19 affect social isolation among people with chronic pain?
	Question 6: Do you sing a song when you are washing your hands? Which song?^#^
Tweet chat 2 (March 25, 2020)	Question 1: What can be done to help people with chronic pain stay out of the hospitals, clinics, and emergency departments?
	Question 2: What is your opinion about telephone and telemedicine consults for patients with chronic pain?
	Question 3*: How is COVID-19 affecting prescriptions and refills of pain medications?
	Question 4: How is physical distancing impacting people with chronic pain?
	Question 5: Are peer support groups being developed by patients during this time of increased stress?

Quantitative data analysis

Quantitative data was summarized in Microsoft Excel using descriptive statistics such as frequency and ranges. Graphical representation of data was generated by Twitter Analytics [7] and Symplur Signals [8]. Twitter Analytics provided the number of impressions, total engagement, engagement rate, likes, replies, and retweets (see Glossary below). Symplur Signals provided the number of tweet activities (number of tweets using #CovidPain per hour), country of tweets, word cloud, sentiment analysis, and healthcare stakeholder segmentation.

Sentiment analysis is a social media analytics tool that involves checking how many negative and positive keywords are present in an excerpt of a conversation. Sentiment analysis was conducted by Symplur Signals. This analysis is powered by a natural language processing (NLP) algorithm that is optimized for healthcare. It extracts subjective information from social media healthcare conversations to determine what is known as the “polarity” - expressed as positive, negative, or neutral. It is a machine learning process for the identification of expressions that reflect the authors’ opinion-based attitudes toward entities [9]. The sentiment analysis widget reveals the most positive and negative tweets. Up to 50 of the most positive and negative tweets are visualized in the graphs [10]. We extracted data for a seven-day period with the date of the tweet chat being the day in the middle. The first tweet chat was extracted from March 15 to 21 and the second from March 22 to 28.

Healthcare stakeholder segmentation was used to categorize the healthcare background of the participants. Symplur Signals uses public data from Twitter account bios and applies machine learning models and algorithms combined with human evaluation and quality control to automatically categorize the top influencers of any given healthcare topic [11]. The categories assigned are as follows: doctor, healthcare professional (HCP), patient advocate, caregiver, researcher/academic, journalist/media, individual health, individual non-health, organization (org.) provider, org. research/academic, org. government, org. advocacy, org. pharmacist, org. MedDevice, org. media, org. other healthcare, org. non-health, spam, and unknown.

Qualitative data analysis

We conducted a qualitative content analysis to examine the narrative material from each tweet that responded to the questions posted. Content analysis is an approach for identifying, analyzing, and reporting themes in rich detail so that we can examine within- and between-group themes and variations [12,13]. COVID-19 is a new phenomenon, and there are no previous studies answering these questions. Therefore, the coded categories were derived directly from the text data following the themes of each predefined question. First, the tweets were downloaded verbatim, and one reviewer (AF) de-identified the tweets by deleting the Twitter handle of the person who wrote the tweet. The user profile information was employed to determine the location and stakeholder group of each tweet. Then, the tweets were copied to a spreadsheet in chronological order. All retweets and replies were removed from this content analysis. Two people (ZD and LB) independently read each tweet and examined its content in relation to answering the tweet chat questions and mapped each tweet to a research question. A tweet could have no relevant content (e.g., greetings) or include multiple relevant answers to one or many questions. In a meeting involving the two coders (ZD and LB) and the principal investigator (AF), all tweets related to each question were grouped into themes. An initial random sample of 100 tweets was used to train the three researchers before the full data analysis. Then, all questions and responses grouped by themes were presented to the other members of this research team. Two rounds of modifications were necessary to the themes, which included adding quotes directly copied from the original tweets. We employed a methodology similar to that of Cheng et al. for analyzing tweet chats on dementia [14].

## Results

Overall participation in each tweet chat

**Figure 1:**
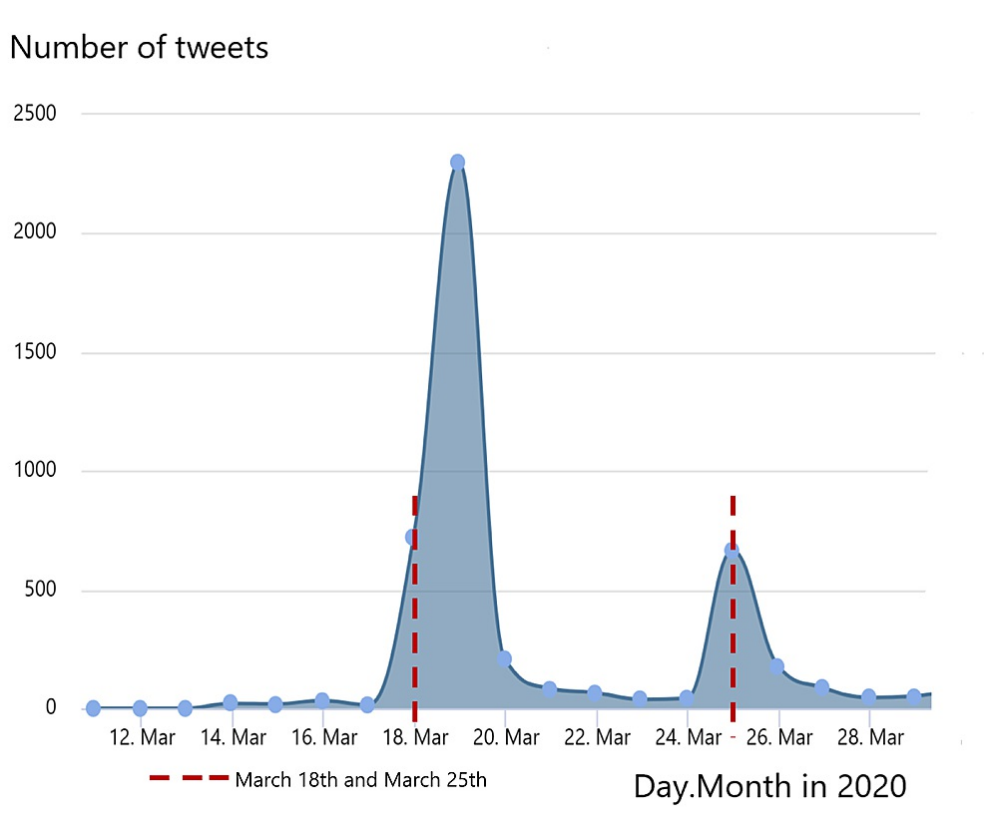
Number of tweets per day mentioning the hashtag #CovidPain from March 11 to April 9, 2020

The first tweet chat included 1978 participants, who contributed 3330 tweets, with an average of 28 tweets per hour. The second tweet chat included 327 participants, who contributed 1021 tweets with an average of nine tweets per hour (Figure 1). In total, there were 14.994 million impressions on Twitter. The countries that contributed with most participants were Canada, the USA, the UK, India, Australia, and South Africa.

The Twitter activity for each research question is shown in Table 2, and it shows that the question that generated the highest impressions (n = 2992) was T1Q2. The question with the highest engagement rate (23.1%) was T1Q5. The question with the lowest overall activity was the dummy question T1Q6. Question T2Q2 performed well for the majority of the evaluation items (impressions, total engagements, engagement rate, likes, replies, and retweets), suggesting that this was the best question to engage with participants. It is important to note that although the number of participants was much smaller in the second tweet chat than in the first tweet chat, the level of participation was comparable among the questions themselves. Moreover, the absolute number of participants did not seem to be associated with the level of participation; for example, the questions with the highest likes and replies were from the second tweet chat, and the questions with the lowest impressions, total engagements, engagement rate, and retweets were from the first tweet chat.

**Table 2:** Twitter activity for each research question Impressions are the number of times a tweet shows up in somebody's timeline. That means every time it's served up, it counts as an impression. Engagements are the total number of times a user interacted with the tweets sent during the selected date range. Likes are when users press the like button represented by a heart. Replies are Tweets directed at the original post. Retweets are people who retweeted the post to their own profile. T1: tweet chat 1; T2: tweet chat 2; Q: question

Question	Impressions	Total engagements	Engagement rate	Likes	Replies	Retweets
T1Q1	2208	117	5.3%	15	7	7
T1Q2	2992	199	6.7%	25	12	11
T1Q3	1893	102	5.4%	12	10	4
T1Q4	1584	109	6.9%	9	4	4
T1Q5	2276	525	23.1%	25	6	6
T1Q6	1589	45	2.8%	6	5	3
T2Q1	2545	271	10.6%	5	18	8
T2Q2	2815	228	8.1%	27	16	8
T2Q3	2235	135	6%	14	7	6
T2Q4	2687	202	7.5%	25	15	5
T2Q5	2090	102	4.9%	13	7	5

Type of healthcare stakeholders involved in each tweet chat

The distribution of the healthcare stakeholders involved in each tweet chat is shown in Table 3. In the first tweet chat, there was a large group of healthcare stakeholders without an identified background (38.2% were unknown stakeholder group), and there was a similar representation from four large groups: healthcare providers, researchers/academics, patient advocates, and doctors. There was also a small proportion of caregivers. In the second tweet chat, the group with the highest representation was doctors, followed by patient advocates and organized advocacy, as well as researchers/academics. All participants in the second tweet chat were known to Symplur Signals and had a healthcare stakeholder background defined.

**Table 3:** Healthcare stakeholders who participated in each tweet chat

Healthcare stakeholders	Tweet chat 1 (n = 1978)	Tweet chat 2 (n = 327)
Individual non-health	1.3%	-
Org. provider	1.3%	-
Org. research/academic	2.6%	-
Caregiver	2.6%	-
Org. advocacy	2.6%	18.2%
Individual other health	5.3%	9.1%
Doctor	6.6%	45.5%
Patient advocate	11.8%	18.2%
Researcher/academic	11.8%	9.1%
Healthcare provider	15.8%	-
Unknown	38.2%	-

Sentiment analysis

**Figure 2:**
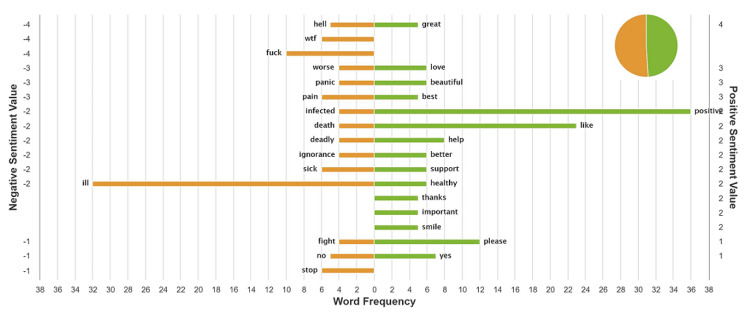
Sentiment analysis for tweet chat 1

**Figure 3:**
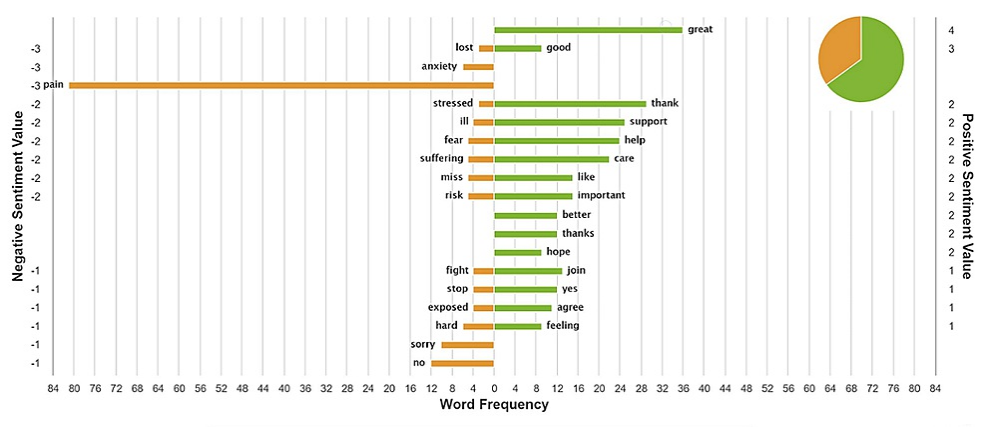
Sentiment analysis for tweet chat 2

The sentiment analysis for each tweet chat is shown in Figures 2 and 3. The top 50 tweets that informed the sentiment analysis for the first tweet chat occurred mainly in March 19, and the results were split almost 50%/50% positive/negative. The top 50 tweets that informed the sentiment analysis for the second tweet chat occurred mostly in March 25, and the majority was positive.

Word cloud

**Figure 4:**
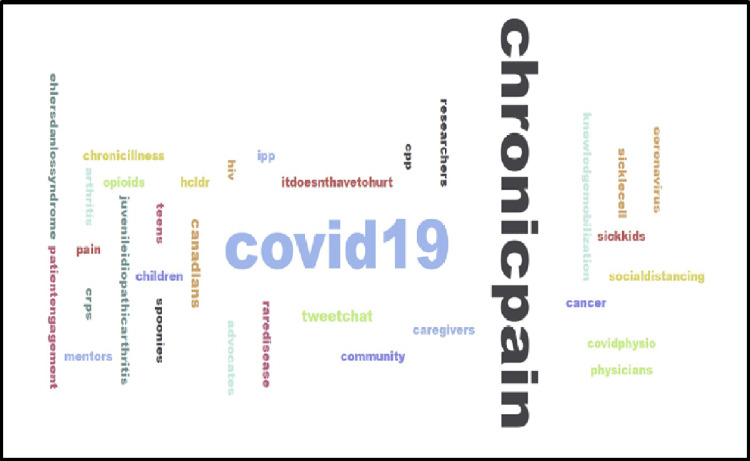
Word cloud of tweet chats #CovidPain

A word cloud was created (Figure 4) with information from both chats. The two most prominent words were "covid19" and "chronicpain." The other words were equally distributed in terms of size. These words suggest that there was representation from various age groups (children and teens), various stakeholders (physicians, researchers, caregivers, community, spoonies, intractable pain patient (IPP), and chronic pain patients (CPP)), and various diseases and conditions (Ehlers-Danlos syndrome, complex regional pain syndrome (CRPS), arthritis, rare diseases, cancer, and sickle cell).

Qualitative content analysis

After removing replies and retweets, there were 1795 unique tweets from the first tweet chat and 568 unique tweets from the second tweet chat. The content of the responses is summarized below.

Tweet Chat 1 Question 1: How Is COVID-19 Affecting Prescriptions and Refills of Pain Medications?

There were four themes in this discussion. The first two were about the negative impact of the pandemic, and the other two were more positive consequences of the pandemic. First, the participants expressed concerns about misinformation regarding the safety of nonsteroidal anti-inflammatory drugs (NSAIDs), namely, ibuprofen, in the progression and susceptibility to COVID-19, as well as how to use pain medications and insurance during the pandemic. Second, the participants expressed their concerns and anxieties about obtaining prescriptions and refills of opioids during the pandemic. Some expressed that they were unable to contact their healthcare professionals due to appointment cancellations. Third, there was optimism in the discussion regarding the potential to expand the scope of practice for pharmacists. Some participants mentioned that Health Canada was considering revising the rules for pharmacies, allowing them to dispense controlled substances without consulting the prescriber. There was also the benefit of the pharmacist’s ability to transfer prescriptions to another pharmacy. Finally, the participants mentioned the beneficial effects of telemedicine to obtain prescriptions, including the ability to bypass lines and waiting rooms by refilling their prescriptions this way, mitigating the need to travel to get their prescriptions.

Tweet Chat 1 Question 2: How Is COVID-19 Affecting the Mood of People With Chronic Pain?

We identified three themes in this discussion. The first was related to the aggravation of depression, anxiety, stress, sleep disorders, isolation, and losing contact with family and friends. The second was the development of new stressors related to homeschooling, worrying about the news, job security, financing, working from home, family crises, and frustrations in general. Third, the participants mentioned various coping mechanisms to handle mood problems, which included self-management, exercises, links to online or community resources, participation in groups, the use of telemedicine to stay connected, and the use of mind-body strategies.

Tweet Chat 1 Question 3: How Is COVID-19 Affecting Physical Activity Among People With Chronic Pain?

Overall, there was an agreement that the maintenance of physical activity was considered an important goal during the pandemic. We identified two themes related to this question, namely, barriers for physical activity and suggestions to remain active during the pandemic. The barriers included gym and physiotherapy clinic closures, financial issues, lack of motivation to exercise, worsening of pain (sometimes due to inability to get their pain medications), and cold outdoor weather. The facilitators included being able to go walking, doing outdoor activities, watching online videos, and practicing mind-body strategies.

Tweet Chat 1 Question 4: How Do Viral Infection Symptoms (Fever and Muscle Aches) Affect People With Chronic Pain?

There were three themes discussed by the participants: uncertainty about using ibuprofen during COVID-19 infection, symptom management, and fear of misdiagnosis of COVID-19 infection. The participants endorsed being unsure about the safety of ibuprofen during COVID-19 infection and mentioned conflicting information and possibly misinformation being spread in the news and social media. They also mentioned that viral infection aggravates preexisting symptoms of pain, fatigue, suffering, anxiety, and worry. There were also concerns about managing viral symptoms in conjunction with chronic inflammatory arthritis, as the medications usually taken for pain have the potential to mask the symptoms of a viral infection. It was also mentioned that COVID-19 may be hard to diagnose as the symptoms could be confused with spring allergies or other respiratory diseases.

Tweet Chat 1 Question 5: How Does COVID-19 Affect Social Isolation Among People With Chronic Pain?

There were five main themes discussed, three of them highlighting the negative effects of social isolation, one about the positive effects, and one about the moral duty of maintaining physical distancing and staying in isolation. First, the participants mentioned that the new rules for social isolation were aggravating preexisting isolation, mental health issues, PTSD, and worries in this population. Some participants endorsed bad experiences with isolation in the past, such as not participating in social or school activities as a result of their chronic pain condition. Second, social isolation during the pandemic created some conflicts at home and family crises; others mentioned loss of contact with some family members. Third, some mentioned the negative effects of social isolation on their daily routines, job issues, financial losses, and adjustment to the new situation. They mentioned that the options for connecting are now limited and that they no longer have small things to look forward to, such as going for a coffee or to a movie. However, some participants mentioned that the isolation provided an opportunity to use new ways of connecting with other people, such as using telemedicine or telephone to contact their healthcare professionals, connect with friends, and even buy and use cannabis. Finally, there were comments that staying isolated is a moral duty to protect healthcare professionals and take care of each other; it is also important to take proper hand hygiene measures, respect social distancing, avoid crowds, and refrain from traveling.

Tweet Chat 2 Question 1: What Can Be Done to Help People With Chronic Pain to Stay Out of the Hospital, Clinics, and Emergency Departments?

There were six strategies that were suggested to keep people safe during the COVID-19 pandemic. First, using virtual care, telephone, emails, or telemedicine to replace face-to-face encounters. The participants suggested that there needs to be investment and government support in telemedicine. They would like to see more virtual meetings with their practitioners, virtual classes of yoga, and myofascial release techniques. Second, the participants mentioned the importance of online support groups for adults and kids, and sharing knowledge about self-management tools. Third, some participants noted the benefits of home induction in the case of buprenorphine/naloxone prescriptions. Fourth, there was a mention of moving care to people's homes and employing more home visits, which are better for patients, for the system, and for the environment. Fifth, some participants talked about the role of pharmacists, especially when they allow longer repeats and greater supplies. Finally, the importance of maintaining preventive measures, especially therapeutic procedures to avoid pain relapse, such as injections for migraines, was discussed.

Tweet Chat 2 Question 2: What Is Your Opinion About Telephone and Telemedicine Consults for Patients With Chronic Pain?

This was the question that had the most sustained engagement during the tweet chats. There were three themes identified in the discussions: challenges of telemedicine, benefits of telemedicine, and suggestions for future changes. The challenges included privacy concerns, technical issues, the fact that telemedicine cannot replace certain aspects of a clinical encounter, difficulty in contacting doctors via email or messaging, or doctors not showing up to the appointment, as well as personal lack of access to Wi-Fi and email. The difficulty in performing physical and examinations, and observing nonverbal cues, e.g., for kids with developmental delays, was also noted. The benefits of telemedicine were many, but mostly, its large-scale implementation was long overdue, and the pandemic accelerated this process. Telemedicine was viewed as good for triage, reassurance, and follow-up, when physical examination is not at all necessary. The benefits included not needing to leave their houses, reduced time in the waiting room, and reduced barriers to access and travel time; therefore, it is most relevant for people living in rural areas. It is important for continuity of treatment, for both physical therapy and mental health, and also useful in renewing prescriptions. Tweet chat participants mentioned that virtual care was better than no care at all. Lastly, tweet chat participants offered suggestions for the future, such as that video conferencing is better than a phone call and a live video is better than a recorded session. They also suggested taking some measures to improve privacy, such as wearing headphones and platforms that are compliant with local privacy regulations. They mentioned that they hope telemedicine will continue past the pandemic, and they hoped that government support will continue.

Tweet Chat 2 Question 3: How Is COVID-19 Affecting Prescriptions and Refills of Pain Medications?

Although this was a repeated question from the previous tweet chat, the themes were slightly different. Five themes were identified in this discussion: COVID-19 is reducing access to medications that people living with pain need, there is a lot of misinformation and confusion about NSAIDs and hydroxychloroquine, the use of telemedicine to get prescriptions and refills of medications, changes in pharmacy operations, and how some people had to be switched to different medications because of COVID-19. First, some people were concerned about the potential unavailability of their medications for lupus or rheumatoid arthritis, namely, hydroxychloroquine, if the medications were found to be effective against COVID-19. Some participants also mentioned that opioids, opioid agonist therapies, and sustained release formulations were back-ordered, which created more fear and anxiety. There were still some complaints about difficulty in finding appointments with their prescribers to refill medications and the lack of access to some medicines and medical professionals, in some cases finding medications for children. Second, there was still confusion about drugs such as ibuprofen, particularly if it could be used in children and infants. There was also misinformation about health insurance plans coverage and the off-label use of hydroxychloroquine, a drug approved in Canada for the treatment of rheumatological conditions. Third, there were comments about the use of telemedicine to get prescriptions, which was seen as a positive impact of the pandemic. For example, telemedicine was seen as critical to have good communication not only between patients and prescribers but also telephone communications between doctors and pharmacies. They mentioned the need to create billing codes for physicians to use virtual care. Fourth, the changes in pharmacy operations that occurred after March 20, 2020, were appreciated; pharmacies were more receptive to phone orders and allowed to refill opioid medications. Fifth, some patients noted that they had to be switched to different medications due to the pandemic. For example, some participants that their biologics switched from monthly to weekly injections, others had infusion times interrupted, and some were forced to switch to self-injectables.

Tweet Chat 2 Question 4: How Is Physical Distancing Impacting People With Chronic Pain?

We identified four themes related to physical distancing: positive aspects, negative aspects, the role of technology, and the specific effects of physical distancing on people with chronic pain. First, the positive aspects involved increased empathy among people, more collaborations, support networks, and taking care of each other, as well as pets. They observed more support to healthcare professionals, hope, and kindness. The negative aspects were mainly related to worsening of mental health, such as worries, anxiety, helplessness, fear, anger, grief, and depression. They also noted an increased sense of isolation, lack of exercises and physical therapy, cancellations of many support groups that increased risk of addiction relapse, missing a sense of normal life, limited access to healthcare, and the need for physical touch/hug. Third, they talked about the role of technology such as online resources, its benefits, and the presence of many support groups online, such as yoga classes and exercises. They acknowledged that technology is not a solution for everyone, especially those who are less technologically savvy, such as the elderly, and they had concerns about privacy. The participants also mentioned the importance of using effective communication between providers and patients. Finally, they talked about some specific topics related to people with chronic pain whose pain is getting worse; that there is a difference in the isolation process among people with pain compared to people who do not have pain; and that they have to learn self-management, use of nonpharmacological treatments, use distractions to cope with pain, find online groups, start exercises, and reduce access to injections and medical professionals.

Tweet Chat 2 Question 5: Are Peer Support Groups Being Developed by Patients During This Time of Increased Stress?

The themes centered around the positive and negative effects and how to maintain these support groups. First, the positive effects were seen as taking care of each other, using creative and innovative methods, using cognitive behavioral therapy (CBT)/meditation online groups, and accessing rural and smaller communities. They appreciated the opportunity to learn from each other and adjust to the new normal, and it was useful to raise awareness and new information. They also talked about the positive effects on mental health, fears, and anxieties, and how they practice self-management. Second, the negative effects of virtual support groups included scams, difficulties finding out which ones are trustworthy, some patients not liking virtual care (patient’s preferences), some who are not technologically savvy, and others who were unsure how to access these support groups. Finally, the participants suggested that these support groups will need financial support after the COVID-19 pandemic to continue their operations.

## Discussion

We analyzed two tweet chat activities conducted in March 2020 about the impact of the COVID-19 pandemic on people with chronic pain, healthcare providers, caregivers, and researchers. We were able to engage people from a wide range of stakeholder groups who shared their main concerns and solutions related to each question posted during the tweet chats. The two tweet chat activities attracted doctors, patient advocates, researchers/academics, healthcare providers, and caregivers, totaling 2305 participants, which generated 4351 tweets and almost 15 million Twitter impressions. During the first tweet chat, the hashtag #CovidPain was trending in Canada. While we advertised mainly to target a Canadian audience, the tweet chats included participants from other countries.

The second question of the first tweet chat and questions two and four of the second tweet chat attracted the most sustained attention and generated a significant number of tweets, retweets, and impressions. Given that these questions deal with mental health, social isolation, and the solution to some of these problems (i.e., the integration and development of telemedicine platforms to help patients), they highlighted the significant challenges that were being faced by the chronic pain community early in the COVID-19 pandemic. The sentiments and content of these tweet chats were both positive and negative, with the second tweet chat showing participants becoming more hopeful, which may suggest that they were getting better adjusted to the situation over time. The more common negative consequences of the COVID-19 pandemic were related to the reduction of physical activity, canceled medical appointments or treatments, and more social isolation beyond what this population is already used to. On the other hand, the participants were able to identify positive consequences of the pandemic, such as the efficient use of telemedicine, innovative methods for self-management programs, and at-home interventions.

In March 2020, when these Tweet chats were conducted, there were no information about the long-term consequences of COVID-19 infection. Now, we know that there are many symptoms that may persist beyond the initial acute phase. There is data showing that 94.9% of patients who acquired COVID-19 experienced at least one post-COVID-19 symptom, including persistent fatigue (82.9%), poor sleep quality (56.3%), anxiety (53.2%), and dyspnea (50%) [15]. These long-term sequelae of COVID-19 were not raised or discussed during the tweet chats.

The type of healthcare stakeholders was defined by a Symplur algorithm that we did not have any control over. During the first tweet chat, there were many participants with unknown backgrounds. This is likely because the hashtag #CovidPain was trending in Canada, which refers to a hashtag-driven topic that is immediately popular at a particular time. This trend might have attracted people who were not directly responding to the questions posted during the tweet chat and still did not have a defined background in Symplur Signals, which accounts for the significant unknown cohort in the first tweet chat. It is possible that people participating in the tweet chat forgot to use the hashtag #CovidPain and therefore were not included in the current analysis. However, if they used the “reply to” or “retweet” feature, they were captured in our analyses. Another limitation of Twitter is that tweets have a limited character count of a maximum of 280 characters; therefore, there was a limited amount of information that could be gathered; for example, when they write about lack of support from the government, we do not know what exactly is lacking.

Tweet chats offer a method of grouping tweets on Twitter. Tweet chats (tweet threads) could not replace thoughtful focus group discussions or in-depth interviews; however, they can draw attention to scholarly work that might otherwise be missed or lost in the deluge of daily publication [16]. One of the benefits of having tweet chats is to engage in social media conversations and generate topics that could be useful for formal focus group discussions.

Both tweet chats were done early in the pandemic, so they do not capture the ongoing developments and opinions of people with chronic pain. The tweet chats were conducted in English and therefore restricted participation to people who were fluent in written English only. The small differences seen between the two tweet chats could be attributed to the differences in the composition of the groups.

We acknowledge that sentiment analysis alone cannot provide an understanding of people’s experiences and what caused them to have a positive or negative attitude toward a topic or discussion [17]. Sentiment analysis is a machine learning process involving the application of natural language processing to the identification of expressions that reflect the authors’ opinion-based attitudes toward entities.

The findings reflect the opinions of not only those who participated in the tweet chats but also those who have access to the Internet via computer or phone. From a health equity perspective, this means that the results may not be representative of users who face barriers in accessing Twitter or the Internet but perhaps also videoconferencing for appointments, and this could further disadvantage those who are already disadvantaged.

## Conclusions

Social media platforms are becoming popular methods to connect with patients and clinicians. There is a wide range of attitudes toward the use of public domain social media “big data” population health research, from enthusiasm, through acceptance, to opposition. We showed that a simple content analysis is well suited for this type of heterogeneous and exploratory material. Since this is an exploratory work in an area where not much is known, this type of analysis is suitable for the simple reporting of common issues mentioned in the data.

The content analysis showed that COVID-19 had both negative and positive impacts on people with chronic pain, caregivers, and healthcare providers. The negative consequences included the reduction of physical activity, canceled appointments and treatments, more isolation, deterioration of preexisting mental health problems, and economic consequences. The positive consequences included efficient use of telemedicine, innovative methods for self-management, and at-home interventions.

The analyses of the two tweet chat activities that occurred early in the pandemic provide insight on the main considerations, both positive and negative, that COVID-19 was having for people with chronic pain. The breadth of input and scope of participation from multiple stakeholders suggests that tweet chats may become a platform to bring hot topics to key participants in moments of uncertainty or rapid change.

## Appendices

Glossary

Engagements: Total number of times a user interacted with a tweet and clicks anywhere on the tweet, including retweets, replies, follows, likes, links, cards, hashtags, embedded media, username, profile photo, or tweet expansion 

Engagement rate: Number of engagements divided by impressions 

Hashtag clicks: Clicks on hashtag(s) in the tweet 

Impressions: Times a user is served a tweet in timeline or search results 

Likes: Times a user liked the tweet 

Replies: Times a user replied to the tweet 

Retweets: Times a user retweeted the tweet 
